# Revealing the Hidden Stress Field and Its Thermal Input Dependence in Femtosecond Laser–Processed Silicon Photonic Chip Substrates via Weak Measurement

**DOI:** 10.1002/nap2.70176

**Published:** 2026-06-26

**Authors:** Wanshou Sun, Baili Qiu, Jiaming Li, Haibing Xiao, Yafei Yu, Qingmao Zhang

**Affiliations:** ^1^ Guangdong Provincial Key Laboratory of Nanophotonic Functional Materials and Devices School of Optoelectronic Science and Engineering South China Normal University Guangzhou Guangdong China; ^2^ Intelligent Manufacturing and Equipment School Shenzhen University of Information Technology Shenzhen China; ^3^ Quantum Science Center of Guangdong‐Hong Kong‐Macao Greater Bay Area Shenzhen‐Hong Kong International Science and Technology Park Shenzhen Guangdong China

**Keywords:** residual stress, silicon substrate, spectrum analysis, ultrafast laser processing, weak measurement

## Abstract

With the widespread adoption of silicon photonic chips in optical–electrical co‐packaging, residual stresses induced by femtosecond laser processing in thick bulk silicon substrates have become a critical factor affecting device performance and reliability. However, existing stress characterization techniques struggle to simultaneously achieve high spatial resolution, high sensitivity, and environmental robustness, leaving the subtle stress fields within the heat‐affected zone largely “hidden.” To address this challenge, we propose a noncontact stress detection method based on frequency domain weak value amplification. The approach leverages the photoelastic effect to convert localized stress into birefringence‐induced phase shifts, which are then read out with high sensitivity via weak measurement. Experiments demonstrate that the method achieves a pascal‐level stress detection limit of 0.37 kPa, reveals a quantitative relationship between laser‐deposited energy and residual stress magnitude, and further resolves the weak stress gradients and local anisotropy within the heat‐affected zone of femtosecond laser–processed silicon samples. Its spatial resolution is nearly an order of magnitude higher than that of confocal laser scanning microscopy. This work provides a new tool for high‐precision, in situ stress monitoring in semiconductor manufacturing.

## Graphical abstract

A novel noncontact method based on weak value amplification enables high‐sensitivity stress mapping of femtosecond laser processed silicon photonic chip substrates. It converts microscale stress into the birefringent phase via the photoelastic effect and reads it out with weak measurement, achieving a 0.37‐kPa detection limit. The technique resolves subtle stress gradients and local anisotropy in the laser‐induced heat‐affected zone.

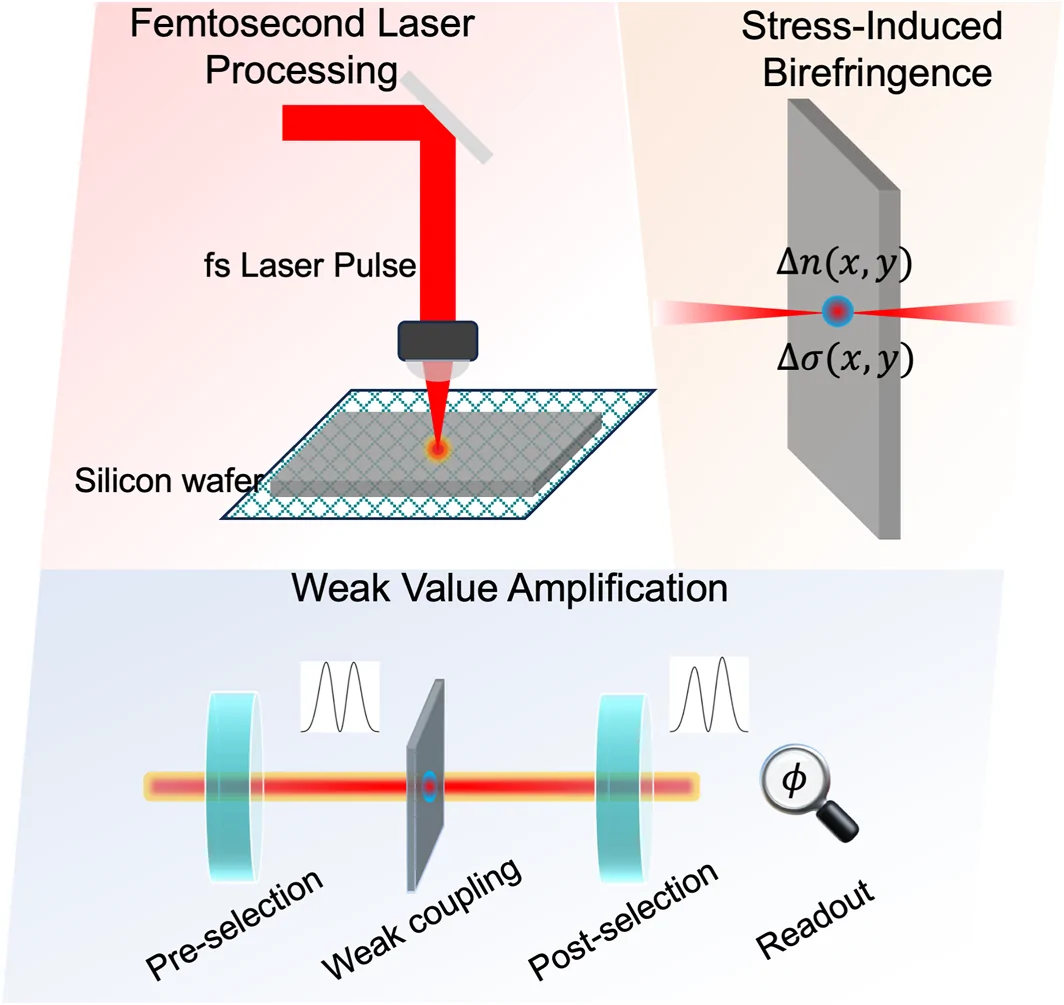

## 1 Introduction

The performance of silicon photonic chips, such as optical modulation efficiency and transmission loss, heavily depends on the stress state of the substrate [1, 2, 3]. In advanced integration architectures like optical–electrical co‐packaging, it is common to fabricate microgrooves and microholes in thick bulk silicon substrates (∼600 μm) using ultrafast laser processing to achieve optical routing and vertical electrical interconnects [4, 5]. Ultraviolet femtosecond lasers have become a key technique for producing high‐precision micro/nano structures due to their “cold” ablation characteristics [6, 7]. However, even at femtosecond timescales, the strong nonequilibrium interaction between laser and silicon induces transient high temperature and pressure conditions along with lattice phase transitions, resulting in significant residual stresses within the heat‐affected zone (HAZ) [8, 9, 10]. Such microscale gradient stresses not only disturb waveguide refractive indices and increase optical losses [11, 12] but also cause frequency drift in micro‐electromechanical systems’ (MEMS’) resonators [13, 14], induce wafer warping, and seriously threaten device performance and packaging reliability [5, 15]. Therefore, developing a technique that can precisely and nondestructively characterize laser‐induced microscale stress distributions is essential for achieving stress engineering and process optimization.

Existing stress detection methods face significant limitations in addressing these challenges: The curvature method based on Stoney's formula lacks sufficient spatial resolution for microscale stress measurements [16]; x‐ray diffraction (XRD) can measure lattice strain [17, 18], but its typical penetration depth does not match the impact depth of laser surface/subsurface processing, and it has low detection efficiency; micro‐Raman spectroscopy, although offering micrometer‐scale spatial resolution [19, 20], is susceptible to sample local temperature fluctuations; traditional photoelastic methods, while useful for visualizing stress [21, 22, 23, 24], struggle to accurately extract stress information under complex background light or material micro‐scattering and absorption; confocal laser scanning microscopy (CLSM) and scanning electron microscopy (SEM) primarily provide topographical information and cannot directly acquire stress field data [10, 25]. These limitations make detecting internal microscale stresses in silicon substrates after laser processing particularly challenging.

To overcome these bottlenecks, exploring new detection principles with high sensitivity and strong environmental robustness is crucial. Recent advances in quantum weak measurement theory, especially techniques based on weak value amplification, provide a new paradigm for ultrasensitive detection of physical quantities [26, 27, 28, 29, 30]. This technology amplifies minute signals (e.g., phase changes) into observable optical pointer shifts and has achieved success in areas such as optical delay/phase [31, 32, 33], beam deflection [34, 35, 36], and refractive index [37, 38] measurements. Although intensity contrast–based weak measurement schemes can resist environmental noise [39], they cannot avoid the effects of material local absorption and scattering inhomogeneity. In contrast, weak measurement schemes based on frequency domain analysis quantify phase changes by analyzing spectral centroid shifts, exhibiting extremely high sensitivity and noise resistance in their linear amplification regime [40, 41, 42]. Although this method faces inherent trade‐offs between sensitivity and dynamic range, recent studies have effectively mitigated these issues through machine learning algorithms [43, 44] and hardware optimizations [45].

In this study, we introduce frequency domain weak value amplification technology into the field of stress detection for silicon‐based laser processing for the first time, successfully establishing a highly sensitive, nondestructive, and interference‐resistant stress characterization platform. We not only achieve precise detection of femtosecond laser–induced microscale stresses but also verify the reliability and unique resolution capabilities of this method in complex microstructure environments through cross‐validation with XRD, SEM, and CLSM. More importantly, using this tool, we reveal the regulation laws of different laser scanning strategies on stress field distribution, providing direct evidence for process optimization. This work not only expands the application scenarios of quantum weak measurement in advanced manufacturing but also opens a new path for stress engineering in high‐integration silicon photonic chips.

## 2 Stress Measurement Theory and Experimental System Setup

### 2.1 Plate Stress Model

The photoelastic effect was first observed by the British physicist David Brewster in 1816 in glass; in 1852, James Clerk Maxwell established the quantitative stress‐optic law to describe this phenomenon [46]. For transparent isotropic materials, the optical properties at any point can be represented by a refractive index ellipsoid (Figure 1a), where the principal axes align with the directions of principal stresses at that point.

**FIGURE 1:**
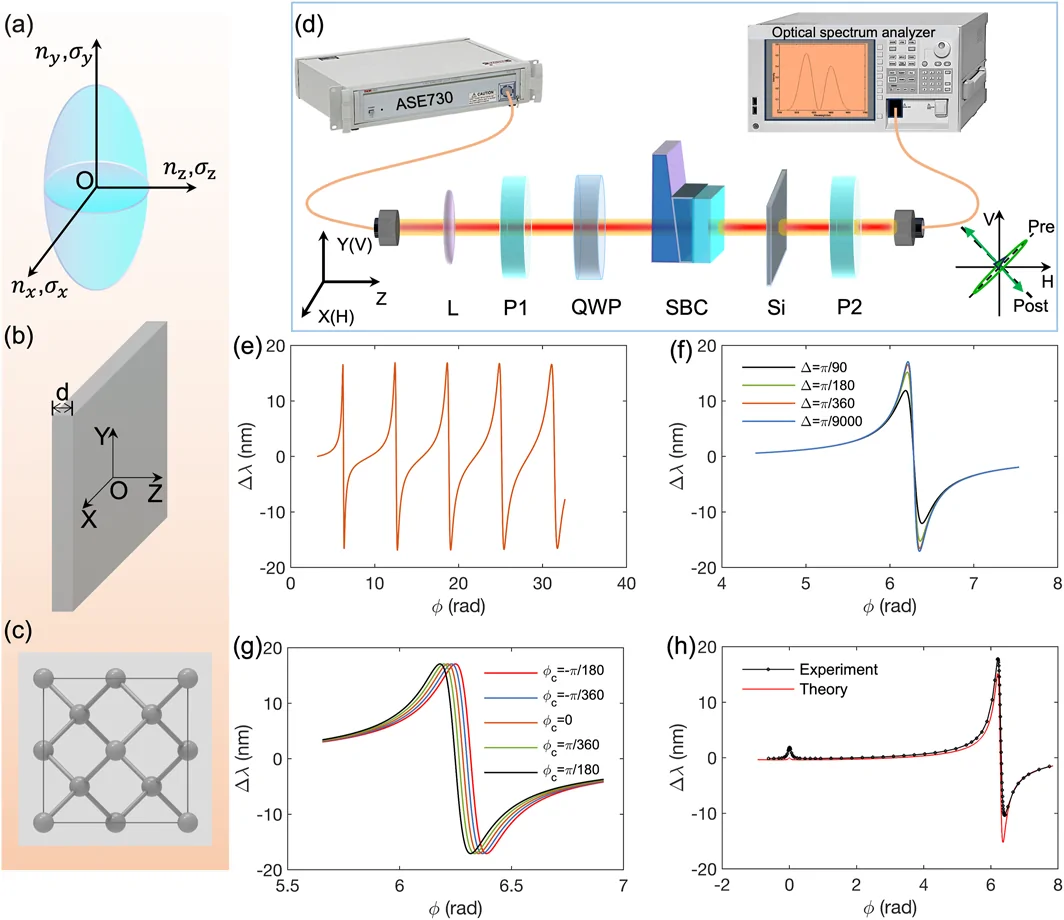
(a) Refractive index ellipsoid aligns with stress principal axes. (b) Schematic of thin plate stress model. (c) Atomic arrangement of the (100) crystal plane in single‐crystal silicon. (d) Experimental setup for stress detection (the inset in the lower right corner shows preselection and post‐selection polarization directions). (e) Periodic relationship between the wavelength shift Δλ and the phase ϕ. (f) Δλ−ϕ curves for different post‐selection angles Δ. (g) Δλ−ϕ curves under varying compensation phases $\phi_{c}$. (h) Theoretical and experimental Δλ−ϕ curves as a function of the phases.

To analyze in‐plane stresses in thin wafer‐like samples, we employ a two‐dimensional plane stress model: Assuming the thin plate lies in the *X*–*Y* plane with zero external force in the thickness (*Z*) direction, the normal stress $\sigma_{z}=0$, while the in‐plane stresses $\sigma_{x}$ and $\sigma_{y}$ remain uniformly distributed throughout the thickness (Figure 1b). Under this condition, the stress‐optic law simplifies to a linear relationship between refractive index differences and in‐plane stress differences:

(1) $$n_{x}-n_{y}=G\left(\sigma_{x}-\sigma_{y}\right),$$

where *G* denotes the material's stress‐optic coefficient, and $n_{x}$ and $n_{y}$ represent the refractive indices in the *x* and *y* directions, respectively.

When a linearly polarized probe beam propagates along the *z*‐direction through a plate of thickness *d*, the orthogonal polarization components accumulate an optical path difference due to birefringence:

(2) $$\delta =\left(n_{x}-n_{y}\right)d=G\left(\sigma_{x}-\sigma_{y}\right)d.$$

The corresponding phase difference is expressed as

(3) $$\phi =\frac{2\pi }{\lambda }\delta =\frac{2\pi }{\lambda }G\left(\sigma_{x}-\sigma_{y}\right)d,$$

where λ is the wavelength of the linearly polarized probe beam. From this, the in‐plane principal stress difference can be derived:

(4) $${\Delta \sigma =\sigma }_{x}-\sigma_{y}=\frac{\phi \lambda }{2\pi Gd}.$$

This relationship is a special case of the three‐dimensional stress‐optics law where $\sigma_{z}=0$. In the general formulation, the refractive index difference vector $B={\left[n_{x}-n_{y},n_{y}-n_{z},n_{z}-n_{x}\right]}^{T}$is proportional to the stress difference vector. However, under the thin‐plate approximation, only the first component dominates the phase modulation for normally incident light.

For (100)‐oriented silicon wafers, the atomic arrangement diagram (Figure 1c) exhibits geometric symmetry. During both laser processing and stress detection, the optical axis is maintained perpendicular to the (100) crystal plane.

### 2.2 Stress Measurement Device and Frequency Domain Weak Measurement Principle

The stress detection apparatus is illustrated in Figure 1d. The system employs an ASE730 broadband light source with a central wavelength of ∼1561 nm and a spectral bandwidth of ∼34 nm. After collimation through a fiber collimator, the light sequentially passes through an aspheric lens (focal length: 250 mm), a polarizer P1, a quarter‐wave plate (QWP), and a Soleil–Babinet compensator (SBC), finally focusing on the sample surface. The sample is mounted on a high‐precision three‐axis translation stage, enabling in‐plane *X*/*Y* displacement and *Z*‐axis rotation for angular alignment. The transmitted beam is coupled into a single‐mode fiber after passing through an analyzer P2, with spectral signals recorded by an optical spectrum analyzer (Yokogawa AQ6370).

Frequency domain weak measurement demonstrates exceptional capability for high‐sensitivity phase detection. We utilize photon polarization as the system's degree of freedom and frequency distribution ω as the probe variable. Assuming a Gaussian spectral distribution for the incident photons, the preselected state $|\psi_{i}\rangle$ and initial probe distribution ϕ(ω) are expressed as

(5) $$|\psi_{i}\rangle =\cos\left(\alpha \right)e^{-i\varepsilon }|H\rangle +\sin\left(\alpha \right)e^{i\varepsilon }|V\rangle ,$$

(6) $$\varphi \left(w\right)=\langle w|\varphi \rangle =\frac{1}{\sqrt[{4}]{2\pi \omega_{0}^{2}}}\exp\left(\frac{{\left(\omega -\omega_{0}\right)}^{2}}{4\sigma_{0}^{2}}\right),$$

where, ω represents the probe frequency variable, $\sigma_{0}$ denotes the probe wave function width, and $\omega_{0}$ is the central frequency of the light source.

The preselection state is prepared using polarizer P1 and QWP: α is the angle between QWP's fast axis and the horizontal direction (set to α=π/4 in experiments), while the angle between P1 and horizontal axis is α+ε, where ε is the preselection angle. The SBC introduces a relative phase shift $2\phi_{c}$ between H and V polarizations, with its fast axis aligned at α+π/4 and a phase compensation range of 0–2π.

In frequency domain weak measurement, the photon polarization and frequency distribution are coupled through weak interaction. When birefringent materials induce a phase shift 2ϕ, the joint system‐probe state becomes

(7) $$\Psi_{j}=(\cos\left(\alpha \right)e^{-i\left(\varepsilon +\phi_{c}+\phi \right)}|H\rangle +\sin\left(\alpha \right)e^{i\left(\varepsilon +\phi_{c}+\phi \right)}|V\rangle |\varphi \rangle .$$

The post‐selected state |$\psi_{f}\rangle$ is defined as

(8) $$|\psi_{f}\rangle =\sin\left(\alpha +\Delta \right)|H\rangle -\cos\left(\alpha +\Delta \right)|V\rangle ,$$

Δ is the post‐selection angle, representing the angle of deviation from α±π/2. Projecting the joint state onto $|\psi_{f}\rangle$ yields the post‐selected probe state as follows:

(9) $$|\phi_{f}\rangle =\left(\left(\cos\left(\Delta \right)+\sin\left(\Delta \right)\right)e^{-i\left(\varepsilon +\phi_{c}+\phi \right)}+\left(\cos\left(\Delta \right)-\sin\left(\Delta \right)e^{i\left(\varepsilon +\phi_{c}+\phi \right)}\right)\right)|\varphi \rangle .$$

Replacing ϕ with $\omega_{0}\tau$, the probe probability distribution becomes

(10) $$\Phi =\langle \phi_{f}|\phi_{f}\rangle =\frac{1}{4}\left[1-e^{-\sigma_{0}^{2}\tau^{2}}\;\cos\left(2\Delta \right)\cos\;2\left(\varepsilon +\phi_{c}+\omega_{0}\tau \right)\right].$$

The post‐selection frequency shift is derived as

(11) $$\Delta \omega =\frac{\langle \phi_{f}\left|\omega \right|\phi_{f}\rangle }{\langle \phi_{f}|\phi_{f}\rangle }-\omega_{0}=\frac{\sigma_{0}^{2}\tau \;\cos\left(2\Delta \right)\sin\;2\left(\varepsilon +\omega_{0}\tau +\phi_{c}\right)}{e^{\sigma_{0}^{2}\tau^{2}}-\cos\left(2\Delta \right)\cos\;2\left(\varepsilon +\omega_{0}\tau +\phi_{c}\right)},$$

Using ω=2πc/λ, the wavelength shift Δλ relates to Δω via

(12) $$\Delta \lambda =\frac{2\pi c\sigma_{0}^{2}\phi }{\omega_{0}^{3}}\frac{\cos\left(2\Delta \right)\sin\;2\left(\varepsilon +\phi +\phi_{c}\right)}{e^{{\left(\frac{\sigma_{0}\phi }{\omega_{0}}\right)}^{2}}-\cos\left(2\Delta \right)\cos\;2\left(\varepsilon +\phi +\phi_{c}\right)}.$$

Substituting Equation (3) into the phase‐stress relationship yields

(13) $$\Delta \lambda =\frac{2\pi \sigma_{0}^{2}\Delta \sigma Gd}{\omega_{0}^{2}}\frac{\cos\left(2\Delta \right)\sin\left(2\left(\varepsilon +\Delta \sigma \text{Gd}\omega_{0}/c+\phi_{c}\right)\right)}{e^{{\left(\frac{\sigma_{0}\Delta \sigma Gd}{c}\right)}^{2}}-\cos\left(2\Delta \right)\cos\;2\left(\varepsilon +\Delta \sigma \text{Gd}\omega_{0}/c+\phi_{c}\right)}.$$

As shown in Figure 1e, the spectral wavelength shift Δλ exhibits a distinct periodic variation with the phase delay ϕ. Within each period, that is, ϕ∈(−π+2nπ,π+2nπ) (n=1,2,3,…), Δλ first increases with ϕ at a gradually increasing slope until reaching a peak, then drops sharply to a trough, and finally recovers with a gradually decreasing slope, entering the next period. When the system satisfies the phase‐matching condition $2\varepsilon +2\phi_{c}+2\phi \approx 2n\pi \;\left(n=1,2,3,\text{…}\right)$, the operating point lies in the high‐sensitivity linear amplification region. In this region, the spectral shift exhibits extremely high sensitivity to phase changes, which is crucial for high‐precision detection of weak stress signals.

The range and sensitivity of this linear amplification region are primarily governed by the post‐selection angle Δ, and the two are inversely related (Figure 1f). Specifically, a smaller Δ yields a larger slope of the response curve and thus higher measurement sensitivity but at the cost of a reduced linear range. Conversely, increasing Δ reduces sensitivity but extends the dynamic range of measurable phases. Therefore, for scenarios involving large stress variations but lower resolution requirements, Δ can be appropriately increased to meet measurement needs.

The compensation phase $\phi_{c}$ is introduced to tune the system's operating point into the linear amplification region. As shown in Figure 1g, varying $\phi_{c}$ translates the Δλ−ϕ response curve without altering the slope (i.e., sensitivity) of the linear region. Leveraging this characteristic, combined with adaptive phase compensation techniques [44, 45], the platform holds promise for extending high‐sensitivity detection to a large stress range. However, to achieve the best measurement precision in this study, all experiments were performed at a fixed operating point with the highest sensitivity (i.e., satisfying $2\varepsilon +2\phi_{c}+2\phi \approx 2\pi$), eliminating the need for dynamic phase control. Excellent agreement between experimental data and theoretical simulations (Figure 1h) fully validates the reliability of the system at this operating point.

It is worth noting that variations in the preselection angle ε produce a superposition of the above two effects: Increasing ε not only leads to a sensitivity reduction similar to increasing Δ but also causes an overall curve translation similar to increasing $\phi_{c}$. In routine experiments, when the signal is sufficiently strong and spectral distortion is absent, both ε and Δ are typically set as close to zero as possible to achieve the highest theoretical sensitivity.

Based on the above principles, we calibrated the key performance parameters of the system. Using the experimental data in Figure 1h, a linear fit of the wavelength shift versus phase delay was performed within the linear amplification region, yielding a system sensitivity of approximately S≈400nm/rad. Combined with the spectrometer resolution of 0.01 nm, the phase resolution of the system was calculated to be $2.5\times 10^{-5}$ rad. Subsequently, using the birefringence formula, Δn=ϕλ/(2πd) (where λ=1561nm and d=0.675mm), the birefringence resolution of the system was derived as ${9.2\times 10}^{-9}$. Finally, incorporating the photoelastic coefficient of a silicon wafer along the (100) direction at 300 K from reference [47], the stress resolution of the system was converted to 0.37 kPa.

## 3 Sample Preparation and Stress Testing

### 3.1 Experimental Planning and Preparation

To achieve precise control and high‐accuracy characterization of residual stress in the processing of silicon photonic chip substrates, this study establishes an integrated experimental framework of “detection–screening–regulation”. Using an 8‐inch (100) silicon wafer as a typical substrate, the radial distribution of residual stress at the wafer edge is first measured by the proposed frequency domain weak measurement stress detection system. Based on the results, a central region with stress fluctuations below ± 0.1 nm is selected and diced into 20 × 20 mm^2^ square pieces, which serve as reference samples for subsequent ultrafast laser processing. The femtosecond laser used in this work (Edgewave, wavelength 343 nm) has a repetition rate adjustable from 400 kHz to 2 MHz, a pulse width of 500 fs, and a single‐pulse energy below 26.5 μJ. To establish the quantitative relationship between heat input and stress, single‐pass single‐scan etching experiments are designed, and SEM morphology analysis is employed to investigate the regulation mechanism of heat input on groove depth and width. Furthermore, for blind hole structures, the proposed method is used to characterize the stress distribution in the heat‐affected zone, while XRD analysis reveals the dynamic response of lattice expansion induced by heat input. Finally, two‐dimensional stress distribution characterization of microstructures such as square etching, blind holes, and through holes preliminarily validates the applicability of this technique to silicon photonic chip substrate processing, providing an experimental basis for subsequent active stress regulation strategies.

### 3.2 Residual Stress Characterization in Bare Silicon Wafers

Before formal sample measurement, reference phase adjustment is performed. Prior to placing the sample, the reference phase $\phi_{c}$ is adjusted by tuning the SBC until the condition $2\varepsilon +2\phi_{c}+2\phi \approx 2\pi$ is satisfied. The averaged spectrum collected under this condition is then established as the reference baseline (${\bar{\lambda }}_{0}$). Subsequently, following the predetermined measurement trajectory and the step size, the sample is moved to the corresponding coordinates (x,y), and the spectrum is scanned again to calculate the mean wavelength (${\bar{\lambda }}_{i}$). The wavelength shift at each measurement point is calculated as $\Delta \lambda_{i}={\bar{\lambda }}_{i}-{\bar{\lambda }}_{0}$, and this shift is directly related to the magnitude of the stress.

Using the above method, we systematically evaluated the stress of an 8‐inch (100) silicon wafer with a thickness of 0.675 mm to verify the high sensitivity and measurement stability of the proposed method and to screen low‐residual‐stress samples suitable for subsequent ultrafast laser processing. The wafer has a surface roughness of less than 0.5 nm, flatness (TIR) < 3 μm, total thickness variation (TTV) < 10 μm, and bow (BOW) < 10 μm, exhibiting excellent surface quality and geometric consistency.

As shown in Figure 2a, a dicing grid on the wafer guides sample cutting. The experiment focuses on the residual stress distribution near the wafer edge, with the thin fan‐shaped silicon piece illustrated in Figure 2b selected as a representative sample. Figure 2c shows a photograph of the actual cut sample, which has a radius of *R* = 15 mm. Stress response data are collected along the OX and OY directions to investigate the stress gradient characteristics induced by edge effects.

**FIGURE 2:**
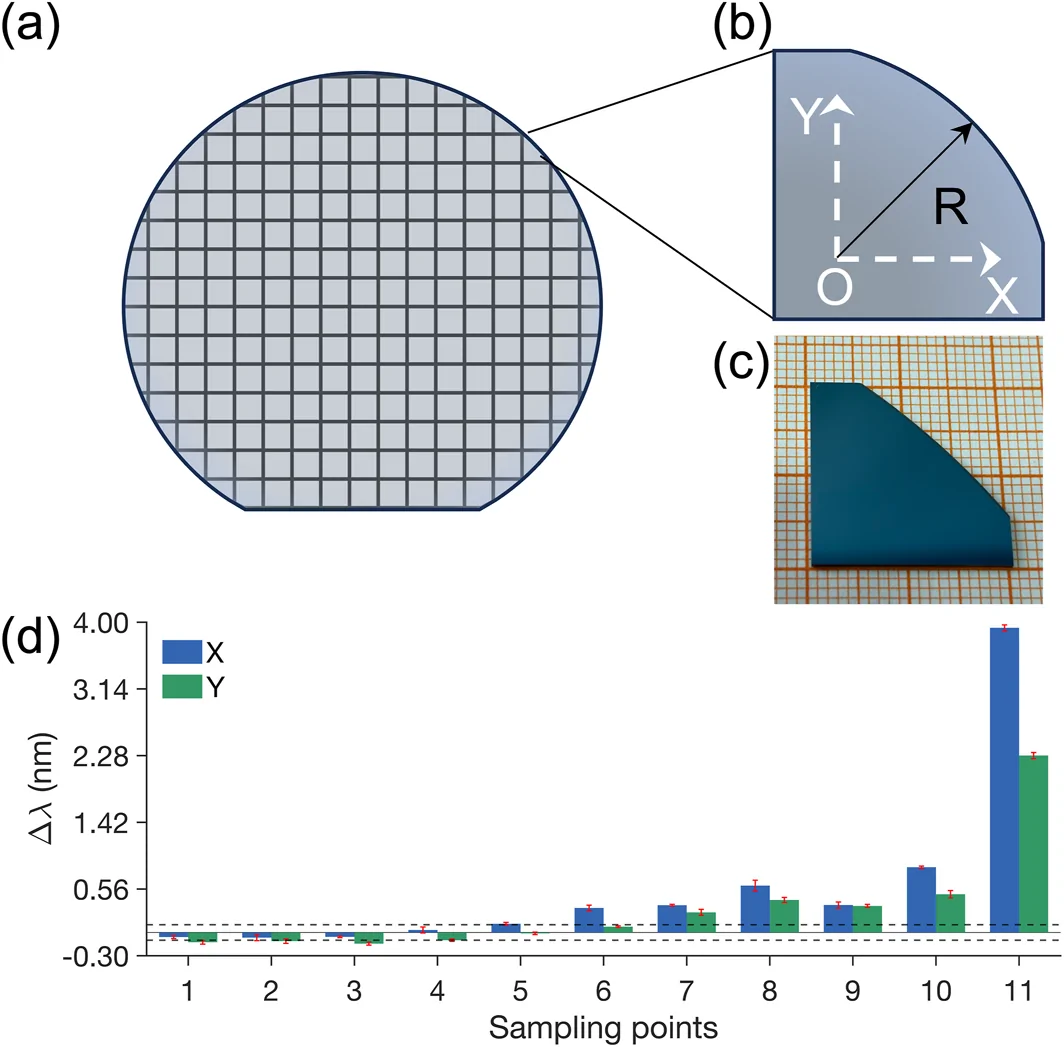
(a) Scribe grid schematic of the 8‐inch silicon wafer. (b) Geometric configuration of the sector‐shaped sample at the wafer edge (*R* = 15 mm). (c) Photograph of the actual cut sector‐shaped sample. (d) Stress responses along *X* and *Y* directions (expressed as average wavelength shifts Δλ). Measurements started from origin O (15 mm from wafer edge) and proceeded along the white dashed arrow paths in (b) with 1‐mm sampling intervals (11 points total). Each point was measured 5 times, with averages and standard deviations calculated (error bars). The upper/lower black dashed lines at Δλ=±0.1 nm correspond to the instrumental wavelength resolution (0.01 nm) multiplied by 10, equating to ±3.7 kPa stress.

As shown in Figure 2d, when the measurement position reached ∼10 mm from the wafer edge (corresponding to the 6th sampling point), stress responses in both *X* and *Y* directions first exceeded the preset ± 0.1 nm threshold, exhibiting a trend of initial increase and gradual decrease, followed by a sharp rise. The average absolute standard deviation across all measurement points was 0.028 nm, within the normal noise level induced by source frequency fluctuations, confirming excellent measurement repeatability.

Based on these observations, to avoid significant stress gradients caused by edge effects, we selected 20 mm × 20 mm square samples from regions farther away from the original wafer edge for subsequent experiments. Each sample underwent five‐point sampling verification to ensure that Δλ fluctuations remained within ± 0.1 nm. This threshold is set to 10 times the instrument's wavelength resolution (0.01 nm), corresponding to a stress value of approximately ± 3.7 kPa. The factor of 10 was chosen as the optimal trade‐off: It ensures that the signal is well above the noise floor while avoiding overly strict rejection of wafers that meet standard manufacturing tolerances, thereby satisfying the experimental requirement for a low‐stress background.

### 3.3 Laser Scribing Experiments

Figure 3a shows a photograph of the silicon wafer after laser scribing. To minimize inter‐sample variations caused by material inhomogeneity, all experiments are performed on the same silicon wafer, which is divided into four independent regions (as shown in Figure 3b and c). To avoid edge effects and mutual interference among processing parameters, laser processing is carried out at the center of each region. Two sets of samples are prepared: one for investigating stress responses under different single‐pulse energies and the other for studying the influence of varying scanning speeds.

**FIGURE 3:**
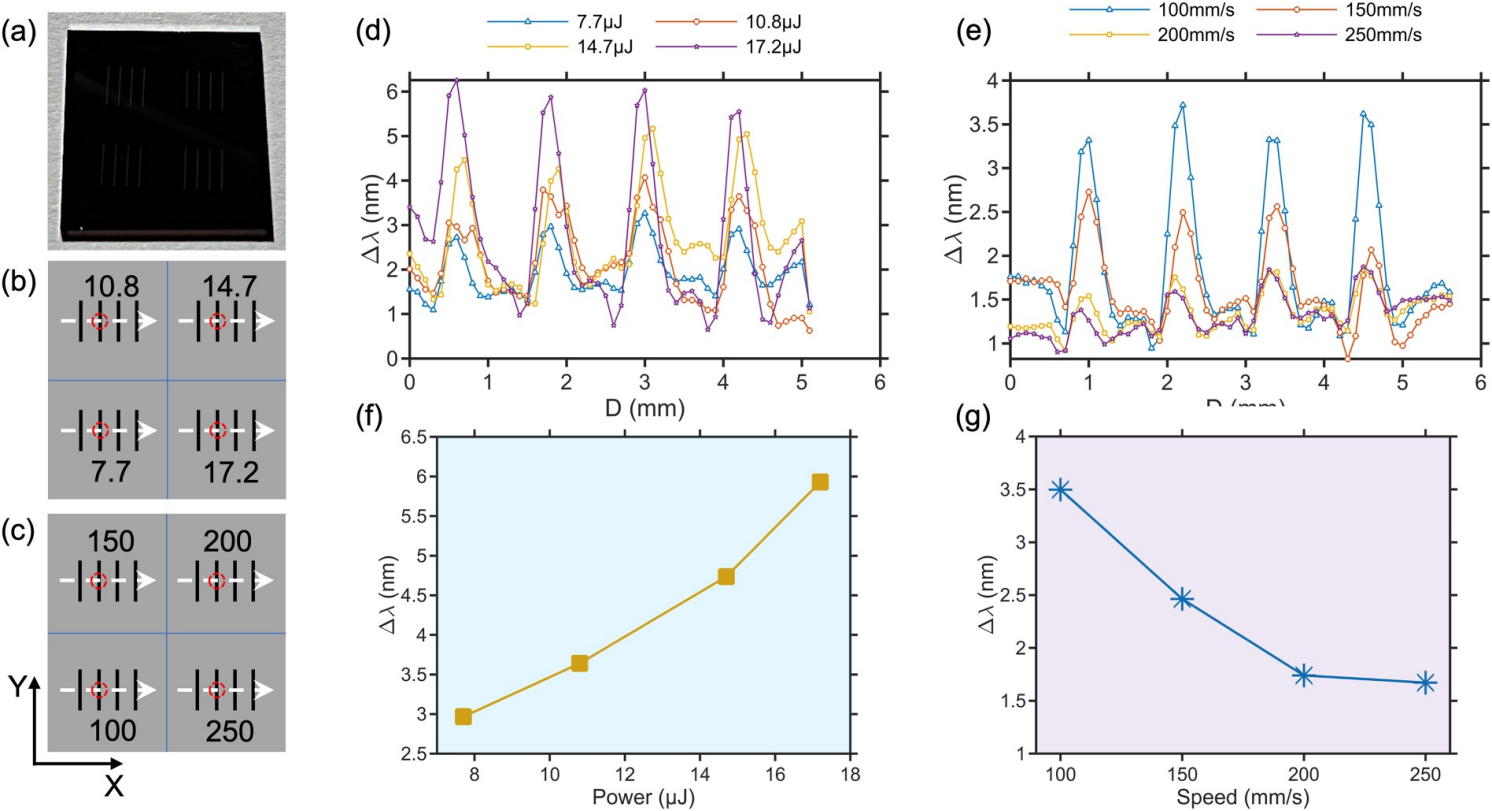
(a) Photograph of the sample after laser scribing. (b and c) Schematic of experimental layout: Gray areas denote four independent test zones; black solid lines represent laser scribing paths (four parallel lines per zone, 4‐mm long and spaced 1.2 mm apart); white dashed arrows indicate *X*‐direction stress measurement paths (100 μm sampling interval); and red dashed circles mark locations for subsequent SEM imaging. (d) *X*‐direction stress response (expressed as Δλ) under fixed scanning speed (200 mm/s) and varying single‐pulse energies (7.7, 10.8, 14.7, and 17.2 μJ). (e) *X*‐direction stress response under fixed single‐pulse energy (10.8 μJ) and varying scanning speeds (100, 150, 200, and 250 mm/s). (f) Average maximum Δλ versus single‐pulse energy. (g) Average maximum Δλ versus scanning speed.

Within each region, four parallel lines are scribed along a predefined path using identical laser parameters. Each line is produced by a single scan, with a length of 4 mm and an inter‐line spacing of 1.2 mm. Stress measurements are conducted along the white dashed arrow paths indicated in Figure 3b and c, with a sampling interval of 100 μm. The results are expressed as wavelength shifts, Δλ.Figure 3d shows the *X*‐direction stress distributions (in terms of Δλ) under fixed scanning speed (200 mm/s) and varying single‐pulse energies (7.7, 10.8, 14.7, and 17.2 μJ). Figure 3e presents the stress responses under fixed single‐pulse energy (10.8 μJ) and different scanning speeds (100, 150, 200, and 250 mm/s).

The maximum Δλ values obtained under each parameter condition (Figure 3f and g) are statistically averaged and plotted against single‐pulse energy (Figure 3f) and scanning speed (Figure 3g), respectively. The results show that the maximum Δλ increases with higher single‐pulse energy, while it decreases with increasing scanning speed. This demonstrates that controlled “writing” of localized residual stress can be achieved by precisely tuning the laser‐deposited energy. The magnitude of induced stress exhibits a positive correlation with energy input—greater energy deposition leads to larger residual stress—confirming the tunability and quantitative relationship between laser energy and stress generation during processing.

Additionally, to investigate the influence of processing parameters on etching morphology, the central segment of the second scribed line in each region is selected for scanning electron microscopy (SEM) imaging, as marked by the red dashed circles in Figure 3b and c. Figure 4a–d show the micromorphologies of etched grooves at different single‐pulse energies (7.7, 10.8, 14.7, and 17.2 μJ) with a fixed laser scanning speed of 200 mm/s. As the single‐pulse energy increases, the laser energy absorbed per unit area of the material rises, leading to a significantly enhanced local heat accumulation effect. Consequently, both the width and depth of the etched grooves increase markedly. The corresponding groove widths are 18.1, 20.0, 24.4, and 25.7 μm, respectively. Figure 4e–h illustrates the evolution of groove morphologies at different laser scanning speeds (250, 200, 150, and 100 mm/s) with a fixed single‐pulse energy of 10.8 μJ. A lower scanning speed results in a longer laser–material interaction time, higher effective energy deposition density on the material surface, and thus larger groove width and depth. The measured groove widths are 16.4, 17.7, 19.2, and 21.7 μm, respectively.

**FIGURE 4:**
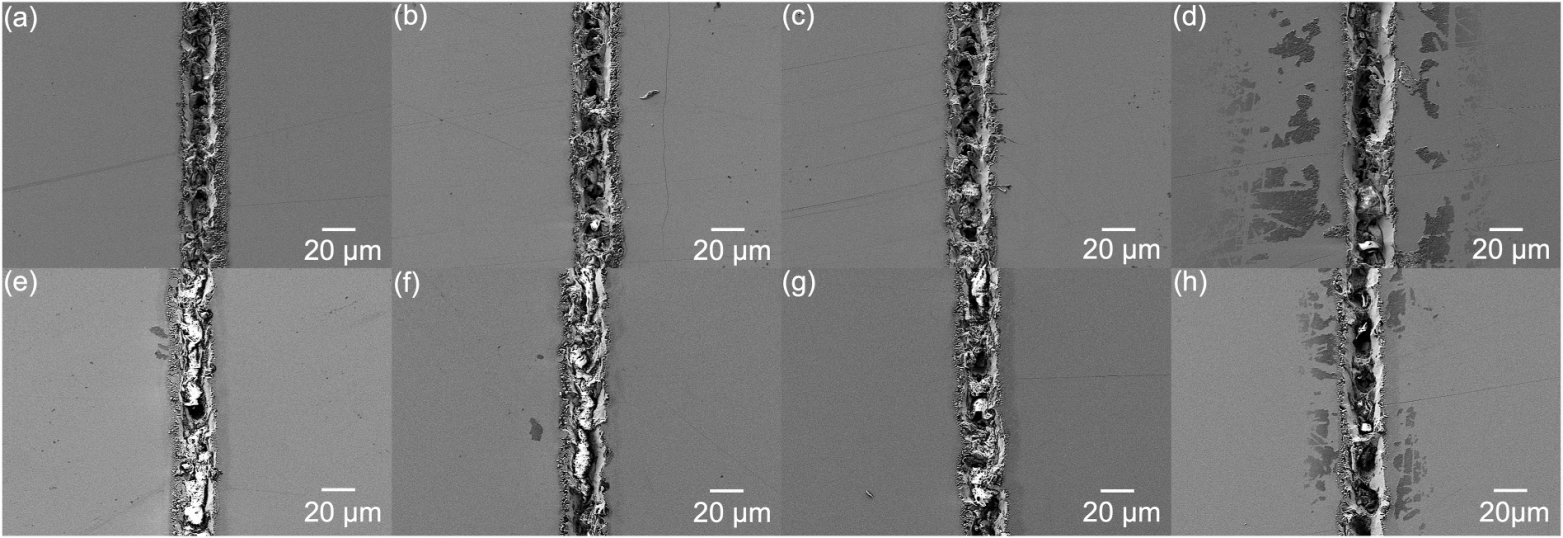
SEM cross‐sectional morphologies of femtosecond laser single‐pass etched silicon wafers under different process parameters. (a–d): Single‐pulse energy variation group (7.7, 10.8, 14.7, and 17.2 μJ) with constant scanning speed of 200 mm/s. (e–h): Scanning speed variation group (250, 200, 150, and 100 mm/s) with constant single‐pulse energy of 10.8 μJ. All images are taken from the regions marked by red dashed circles in Figure 3b and c. Scale bar: 20 μm.

In summary, both increasing single‐pulse energy and reducing scanning speed enhance local energy density, intensifying thermal effects and leading to more pronounced material removal and structural deformation. This morphological enhancement is closely linked to greater thermal accumulation, which likely induces stronger thermomechanical stress responses. This trend aligns well with the observed dependence of Δλ on energy and speed in Figure 3d–g, further validating the positive correlation between energy input and residual stress.

It should be noted that surface characterization techniques such as SEM cannot probe internal stress states, whereas the performance stability of silicon photonic devices is often governed by subsurface residual stresses. The frequency domain weak measurement technique employed in this work enables nondestructive, in situ extraction of internal stress distributions through high‐sensitivity detection of wavelength shifts (Δλ), thereby establishing a direct link between surface morphology evolution and internal mechanical response. This provides a solid foundation for stress engineering in ultrafast laser micromachining.

### 3.4 Effects of Laser Scanning Strategies

In the realm of laser processing on single‐crystal silicon, existing research predominantly focuses on the directional dependence of stress responses due to crystal orientation itself [47, 48]. However, in applications such as MEMS device fabrication, the symmetry of the stress field introduced by laser processing strategies is equally crucial, directly affecting device performance and reliability. Clarifying the relationship between process parameters and stress anisotropy is key to achieving precise stress control and optimizing microstructure functionality.

To systematically investigate the influence of the laser scanning strategy on the stress distribution in single‐crystal silicon, we designed a series of two‐dimensional microstructured samples with controllable laser scanning speeds and processing passes. Their photographs and layouts are shown in Figure 5a–c. The sample surface contains four groups of laser ablation regions, corresponding to the variable scanning speed experiment (Figure 5b) and the variable processing pass experiment (Figure 5c), respectively. All ablation regions have an area of 2 mm × 2 mm and are spaced 6 mm apart. Using the established stress measurement system, equispaced measurements with a step size of 200 μm were performed along the white dashed paths (Paths 1–8) marked in Figure 5b and c. The results are shown in Figure 5d and e, respectively.

**FIGURE 5:**
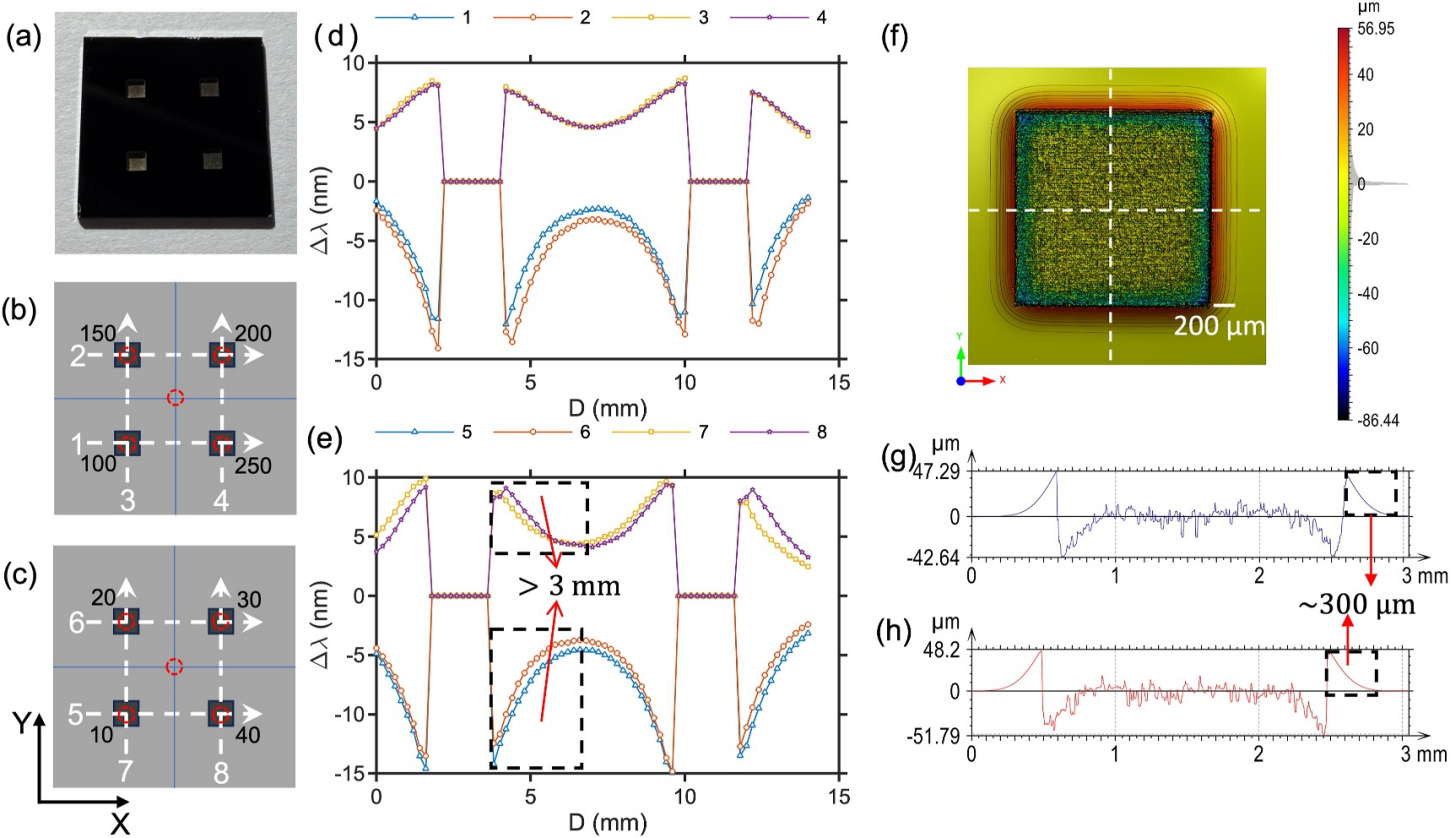
(a) Photograph of the sample after laser etching (variable scanning speed group). (b) Layout of the variable scanning speed experiment: The four black regions are ablation areas, corresponding to 100, 150, 200, and 250 mm/s in counterclockwise order starting from the bottom‐left; white dashed arrows indicate the stress measurement paths (Paths 1–4). In this group, the number of scanning passes is fixed at 10, and the single‐pulse energy is 10.7 μJ. (c) Layout of the variable processing pass experiment: The ablation areas correspond to 10, 20, 30, and 40 passes in counterclockwise order starting from the bottom‐left. Paths 5–8 are defined in the same manner. In this group, the scanning speed is fixed at 200 mm/s, and the single‐pulse energy is 14.7 μJ. (d) Stress response curves along the *X* and *Y* directions at the center of the ablation areas under different scanning speeds. (e) Corresponding stress response results under different numbers of laser processing passes. (f) Two‐dimensional confocal microscopy topography of the region processed with 10 passes. (g and h) Profile contours extracted along the center lines in the *Y* and *X* directions, respectively, showing a heat‐affected zone width of approximately 300 μm. The red circles in (b) and (c) mark the positions for subsequent XRD measurements; the central area was not subjected to laser processing.

The stress distribution profiles in Figure 5d and e indicate that the stress amplitude gradually increases as the measurement position approaches the ablated region, and the growth rate accelerates near the edge. Due to the high surface roughness of the ablated region, the incident light is strongly scattered and cannot penetrate, making it impossible to collect valid spectral information; therefore, the stress in this region is uniformly set to zero. Although variations in processing parameters cause nonlinear fluctuations in stress amplitude, the stress distribution consistently exhibits significant anisotropy: The stress amplitudes along the *X*‐direction paths 1, 2, 5, and 6 are markedly larger than those along the *Y*‐direction paths 3, 4, 7, and 8. Specifically, under different scanning speeds (Figure 5d), the ratios of the maximum stress amplitude in the *Y*‐direction to that in the *X*‐direction are 69.6% (100 mm/s), 74.0% (150 mm/s), 64.1% (200 mm/s), and 60.3% (250 mm/s), with an average value of 67.0%; under different processing passes (Figure 5e), the ratios are 64.4% (10 passes), 61.5% (20 passes), 63.1% (30 passes), and 66.3% (40 passes), with an average value of 63.8%. This pronounced and reproducible anisotropy (with the *Y*‐direction stress amplitude being on average ∼35% lower than the *X*‐direction amplitude, based on the average *Y*/*X* ratios of 67.0% and 63.8% for the two parameter groups) confirms that the processing strategy is the dominant factor governing the stress anisotropy. As a control, the residual stress fluctuation in an unprocessed pristine (100) silicon wafer is only within ± 0.1 nm (equivalent Δλ) in both *X* and *Y* directions, which is far smaller than the differences observed after processing, thereby ruling out the influence of initial material anisotropy.

We believe that this stress anisotropy directly stems from the adopted zigzag scanning path. In this strategy, the laser beam scans parallel to the *X* direction but requires deceleration, turning, and acceleration processes at the start and end points of each scan line (corresponding to boundary regions in the *Y* direction). This process prolongs the interaction time between the laser and material in these regions, resulting in higher local energy deposition and more intense thermal cycling, ultimately inducing greater residual stress at the *Y* direction boundaries. Our findings highlight that scanning strategy is a critical process variable for controlling the stress symmetry in laser‐processed silicon microstructures.

Notably, the frequency domain weak measurement method proposed in this paper is capable of detecting elastic strain fields that extend far beyond the scale of surface topography variations. To visually verify this, we performed confocal microscopic topography characterization on the region with 10 laser scanning passes in Figure 5c (see Figure 5f) and extracted the surface profiles along the centerlines in the *Y* and *X* directions (Figure 5g and h). The width of the HAZ, defined by abrupt height changes, is only about 300 μm (indicated by the black dashed box in the figure). In contrast, the stress response decay span measured by our method at the same location exceeds 3 mm (black dashed box in Figure 5e). This significant difference in scale (an order of magnitude) strongly demonstrates that the laser‐induced residual stress field extends far beyond the topographically visible damaged region and that our method can effectively characterize such a wide range of subsurface stress distribution.

XRD is an important technique for detecting internal residual stresses in materials with crystalline structures. When the surface layer of a material is subjected to tensile stress, lattice spacing increases; according to Bragg's diffraction principle (2dsinθ=nλ), corresponding diffraction peaks shift toward lower angles. In this study, XRD tests were performed on the aforementioned samples, with results shown in Figure 6. Compared to unprocessed areas, all characteristic diffraction peaks shifted to lower angles to varying degrees with increased laser thermal input, showing a positive correlation trend with thermal input intensity. Among them, the diffraction peak near 69.1° corresponds to the Si (400) plane, whose normal direction is [100], making it the most direct and sensitive diffraction peak for detecting in‐plane stress on (100)‐oriented single‐crystal silicon surfaces. Tensile strain on the surface layer causes an increase in the (400) plane spacing, leading to a shift of the 2θ angle toward lower angles. Additionally, the diffraction peak observed at approximately 32.8° belongs to the Si (200) plane, which is the second harmonic of the (400) plane, hence exhibiting strong diffraction intensity. Notably, this peak also shows a clear shift toward lower angles, with the degree of shift increasing with enhanced laser thermal input. This phenomenon suggests that the lattice expansion induced by laser processing is not limited to specific crystal planes but rather arises from overall thermal effects causing widespread lattice distortion.

**FIGURE 6:**
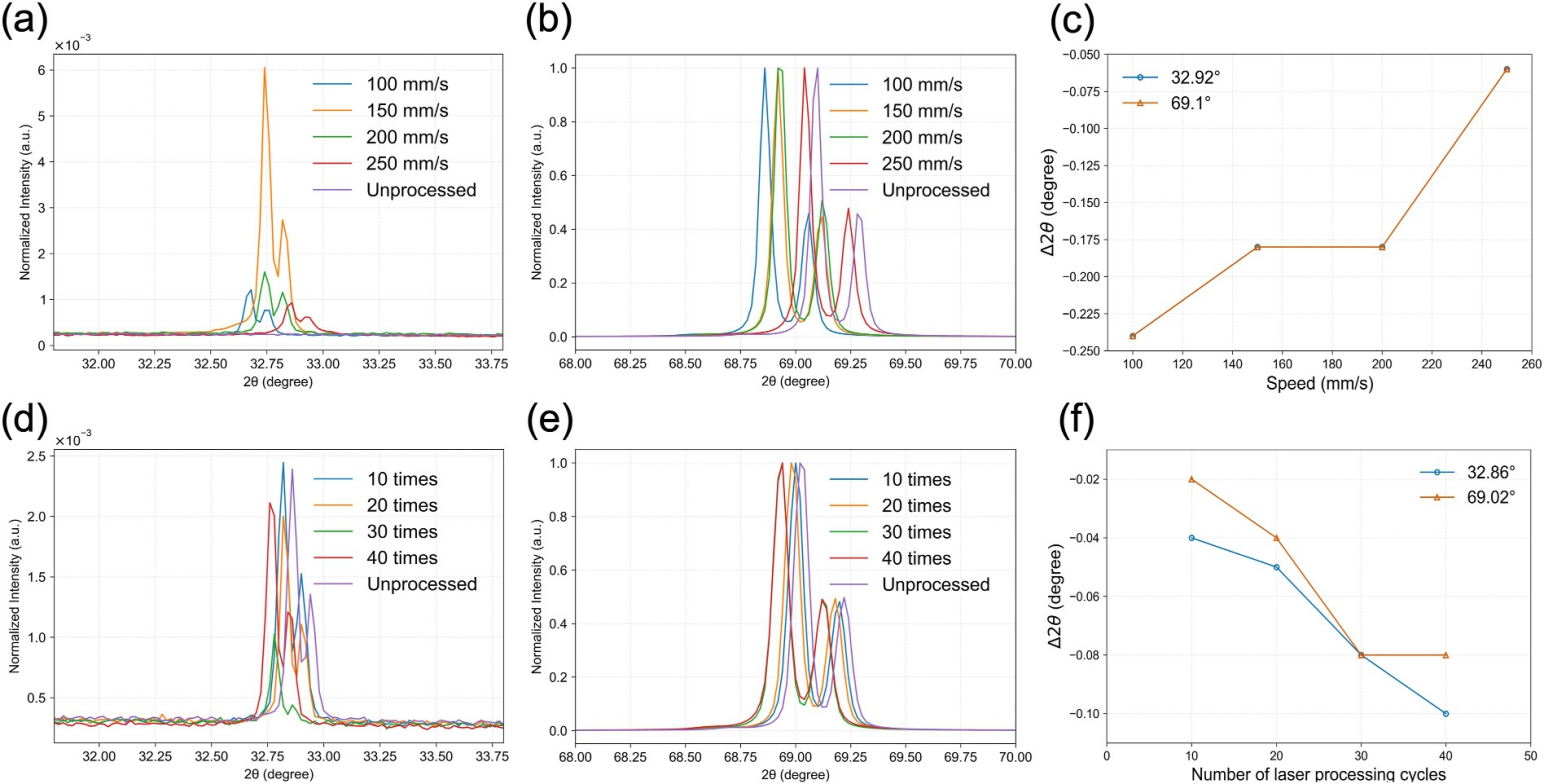
XRD analysis results of residual stresses on the surface of laser‐processed single‐crystal silicon. (a) Diffraction peaks of the (200) plane in blind hole regions and unprocessed areas under different scanning speeds. (b) Diffraction peaks of the (400) plane under different scanning speeds. (c) Shifts in diffraction peak positions (Δ2θ) of the (200) and (400) planes as a function of laser scanning speed. (d) Diffraction peaks of the (200) plane in blind hole regions and unprocessed areas under different laser ablation cycles. (e) Diffraction peaks of the (400) plane under different laser ablation cycles. (f) Shifts in diffraction peak positions (Δ2θ) of the (200) and (400) planes as a function of laser ablation cycles. Labels in (c) and (f) indicate the initial 2θ values of diffraction peaks in unprocessed areas, used for quantifying peak position shifts.

It should be noted that theoretically, no diffraction peak for the (200) plane should be observed in ideal single‐crystal silicon (due to zero structure factor). However, the “(200)” signal detected near 32.8° during experiments does not stem from intrinsic diffraction behavior of complete crystals but is likely caused by localized lattice disorder, defect structures, or multiple scattering effects introduced during laser processing. This further indicates that laser treatment alters the local crystal integrity of the material, allowing previously forbidden diffraction channels to appear.

By analyzing the angular shifts of diffraction peaks (Δ2θ), the magnitude of changes in internal residual stresses can be quantitatively calculated. Figure 6c and f show that with increasing laser thermal input, both characteristic diffraction peaks exhibit continuous shifts toward lower angles, consistent with the trend observed in previous single‐pass experiments where higher thermal inputs resulted in greater stresses. If a two‐dimensional grid scan were performed on the sample, spatial distribution information of in‐plane stresses could be obtained. However, traditional XRD methods have notable limitations in terms of time cost and efficiency for large‐area stress characterization due to the long duration required for single‐point XRD measurements.

Notably, the stress detection method proposed in this study is limited by high surface roughness in the ablation region, causing intense light scattering and preventing reliable stress data acquisition in this area. Therefore, direct comparisons with XRD results within the ablation region cannot currently be made. Nevertheless, the stress evolution trends reflected in measurable regions (i.e., flatter surface areas) by both methods are highly consistent, validating the effectiveness of the stress characterization method used in this study.

Furthermore, XRD measurements themselves are susceptible to surface morphology influences. Rough surfaces may alter diffraction geometry conditions (such as deviations in incident and reflection angles) and lead to systematic angular errors, causing overall shifts or broadening of diffraction peaks. Surface roughness also modifies the effective penetration depth and absorption path of X‐ray beams, affecting the intensity distribution of diffraction peaks, especially in low‐intensity diffraction peaks. These effects are not due to real lattice distortions but arise from measurement condition changes leading to “pseudo peak shifts.” Due to current experimental constraints, we did not systematically study the specific response patterns of peak shifts under different roughness levels; related mechanisms will be further explored in subsequent work.

In summary, when performing stress detection on laser‐etched samples, the light scattering and absorption effects introduced by the etched region must be fully considered, especially in scenarios where transmitted or reflected light intensity is used as the probe signal. Although existing multi‐angle polarimetry can achieve this decoupling under both far‐field and near‐field conditions [49, 50], it significantly increases the complexity of the experimental procedure. In contrast, the frequency domain weak measurement scheme proposed in this paper uses the spectral centroid shift as the probe signal, achieving an intrinsic decoupling between the signal and the absorption background at the sensing mechanism level. Under this mechanism, the absorption and scattering of the material are merely regarded as common‐mode noise that does not affect the shape of the spectral distribution. This allows us to extract effective information directly from a single spectrum and obtain the stress state of the sample without the need for complex post‐processing algorithms.

### 3.5 Surface Stress Detection

To further explore the distribution characteristics of residual stresses in silicon‐based micro‐mechanical structures, we designed and fabricated three typical geometries: surface etching, blind holes, and through holes, which were processed using ultraviolet femtosecond lasers. Figure 7a–f show photographs of these structures and their processing layouts. All samples were processed under identical laser parameters: repetition rate of 600 kHz, single‐pulse energy of 14.7 μJ, and scanning speed of 500 mm/s. For blind holes and through holes, a zigzag scanning strategy was employed with a line spacing of 20 μm to achieve uniform filling. Subsequently, our proposed stress detection system was used to perform two‐dimensional grid scanning measurements (with a sampling step of 1 mm) on each sample to obtain the spatial distribution of in‐plane residual stresses. The results are shown in Figure 7g–j. It is important to note that this larger sampling step leads to some spatial offset between the stress distribution map and the actual processing area. Additionally, due to the finite size of the laser spot, the measured signal near the boundary between the ablation zone and the HAZ represents a superposition of responses from both the ablated region and its surrounding thermally influenced area.

**FIGURE 7:**
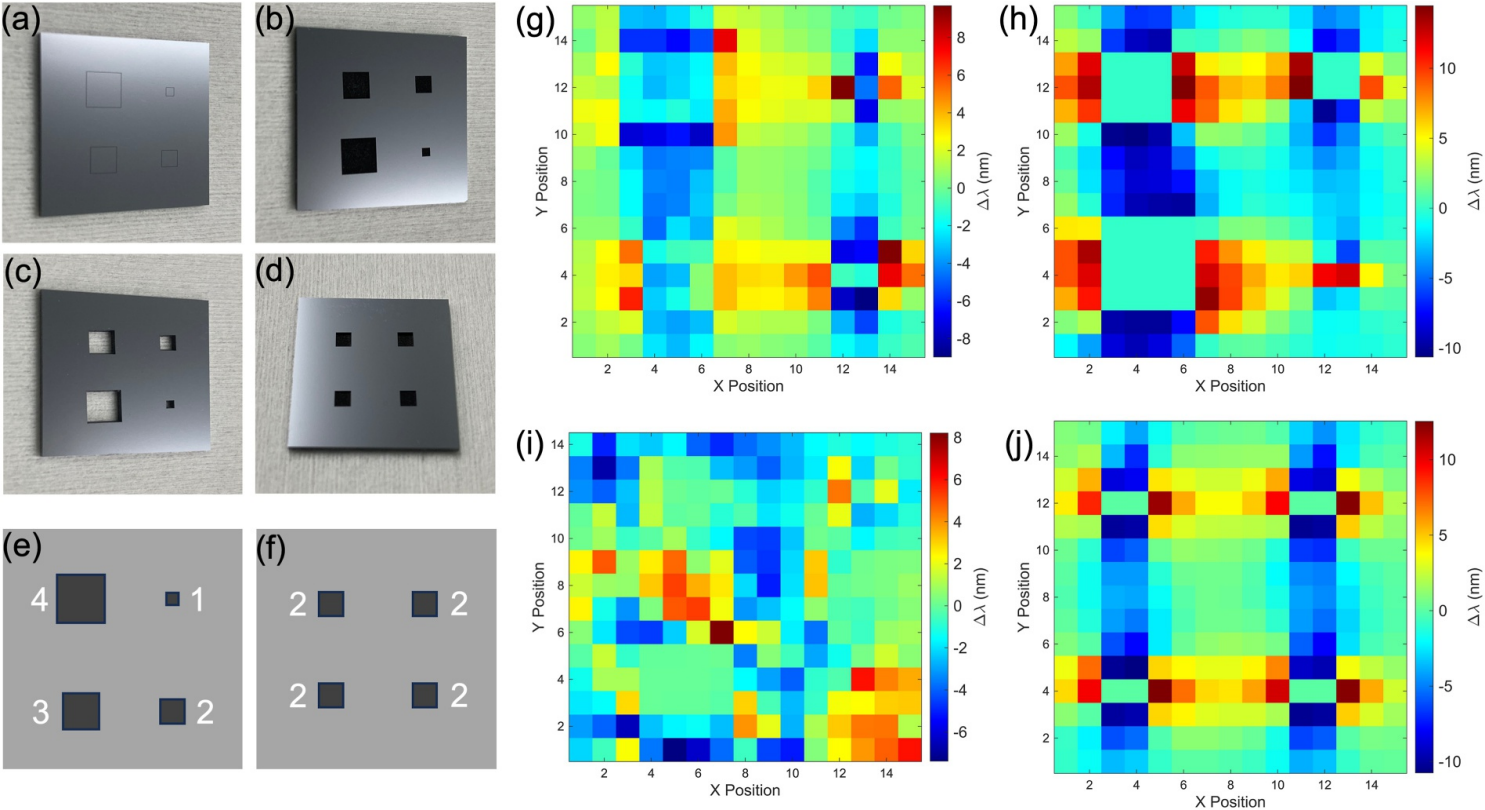
(a) Photograph of a single surface etching structure, where only edge scribing along a square boundary was performed without filling. (b) Photograph of a blind hole structure processed under the same parameters as (a) but with 10 repeated scans. (c) Photograph of a through hole structure processed under the same parameters as (a), with over 80 scan repetitions. (d) Photograph of four blind holes arranged in a 2 mm × 2‐mm square area, using a zigzag scanning strategy with a line spacing of 20 μm. (e) Schematic diagrams of the structures in (a–c), indicating dimensions of 1 mm, 2 mm, 3 mm, and 4 mm square regions. (f) Schematic layout of the four blind holes in (d). (g–j) Two‐dimensional grid scanning stress distribution maps corresponding to (a–d), respectively, with a sampling step of 1 mm. Colors represent stress variations (Δλ) in nm.

However, because the ablation zone has high surface roughness, incident light undergoes intense scattering in this region, making it difficult to extract reliable stress signals. In contrast, the lattice distortion within the HAZ is more continuous, allowing its stress state to be effectively detected using noncontact optical methods. Therefore, when analyzing the stress distribution near the edges of the ablation zones, it is essential to consider the coupling effects of the scanning step and the spot size to avoid misinterpretations caused by insufficient spatial resolution or signal aliasing.

Despite these limitations, the experimental results clearly reveal the influence of different microstructure geometries on the stress field: The surface etching region (Figure 7g) shows stress concentrations at the edges, exhibiting localized high values; the blind hole structure (Figure 7h, j), due to material removal, releases stress and forms a ring‐shaped gradient distribution; the through hole structure (Figure 7i) exhibits a more complex multidirectional stress field, reflecting the impact of depth penetration on three‐dimensional stress reconstruction. These differences demonstrate that processing paths, material removal depths, and geometric boundary conditions collectively determine the spatial evolution of residual stresses, providing critical experimental evidence for the design and optimization of stress engineering in MEMS devices.

## 4 Conclusion and Outlook

This study introduces frequency domain weak measurement into the quantitative characterization of residual stresses in single‐crystal silicon induced by femtosecond laser processing. By detecting stress‐induced minute wavelength shifts (Δλ), we achieve high‐sensitivity, nondestructive mapping of micrometer‐scale stress gradient fields and directly probe the post‐processing “internal stress state.” Notably, the detectable extent of the thermally affected zone significantly exceeds that revealed by confocal microscopy–based surface morphology analysis. Experiments uncover a pronounced influence of laser scanning strategies on anisotropic stress responses in single‐crystal silicon, with the *Y*‐direction stress being approximately 35% lower than the *X*‐direction stress on average. By comparing two‐dimensional stress distributions across microstructures—including scribed lines, blind holes, and through holes—we elucidate how geometry governs local stress confinement versus relaxation. Furthermore, a clear quantitative relationship is established between laser‐deposited energy and the magnitude of residual stress, providing direct experimental guidance for process optimization. These findings hold significant implications for the fabrication and reliability assessment of silicon‐based devices. As devices continue to scale down to micro/nanoscale dimensions and adopt three‐dimensional integration architectures, residual stresses from processing have become a critical factor affecting performance stability and long‐term reliability—particularly in high‐precision or harsh operational environments, where stress evolution may trigger performance drift or catastrophic failure. Looking forward, this stress‐mapping methodology could be integrated with deep elastic strain engineering [51, 52, 53] to enable precise control of elastic strains in single‐crystal silicon, thereby facilitating systematic investigations into strain‐dependent physical properties. Such capabilities would provide a powerful foundation for designing next‐generation silicon‐based optoelectronic devices and functional microstructures. Moreover, as material characterization, micro/nanofabrication, and device physics converge ever more closely, this technique holds broad potential for expansion into silicon photonics, MEMS, and advanced heterogeneous integration systems.

## Author Contributions

**Wanshou Sun:** investigation, validation, writing – original draft, visualization, conceptualization, methodology, software, data curation. **Baili Qiu:** software, validation, investigation, data curation. **Jiaming Li:** writing – review and editing, validation, formal analysis, conceptualization, supervision, resources, methodology. **Haibing Xiao:** conceptualization, formal analysis, data curation, resources, supervision. **Yafei Yu:** writing – review and editing, project administration, resources, funding acquisition, supervision, methodology, conceptualization, formal analysis. **Qingmao Zhang:** writing – review and editing, project administration, resources, funding acquisition, supervision, conceptualization, methodology, formal analysis.

## Conflicts of Interest

The authors declare no conflicts of interest.

## Data Availability

The data that support the findings of this study are available from the corresponding author upon reasonable request.
